# Familiar size affects the perceived size and distance of real objects even with binocular vision

**DOI:** 10.1167/jov.21.10.21

**Published:** 2021-09-28

**Authors:** Margaret V. Maltz, Kevin M. Stubbs, Derek J. Quinlan, Anna M. Rzepka, Jocelyn R. Martin, Jody C. Culham

**Affiliations:** 1 Department of Psychology, University of Western Ontario, London, Ontario, Canada; 2 Brain and Mind Institute, University of Western Ontario, London, Ontario, Canada; 3 BrainsCAN, University of Western Ontario, London, Ontario, Canada; 4 Department of Psychology, Huron University College, London, Ontario, Canada; 5 Neuroscience Program, University of Western Ontario, London, Ontario, Canada

**Keywords:** familiar size, distance perception, real-world, binocular vision, 3D vision, object recognition

## Abstract

Although the familiar size of real-world objects affects size and distance perception, evidence is mixed about whether this is the case when oculomotor cues are available. We examined the familiar size effect (FSE) on both size and distance perception for real objects under two viewing conditions with full or restricted oculomotor cues (binocular viewing, which provides vergence and accommodation cues, and monocular viewing through a 1-mm pinhole, which removes those cues). Familiar objects (a playing die versus a Rubik's cube) were manufactured in their typical (1.6-cm die and 5.7-cm Rubik's cube) and reverse (5.7-cm die and 1.6-cm Rubik's cube) sizes and shown at two distances (25 cm versus 91 cm) in isolation. Small near and large far objects subtended equal retinal angles. Participants provided manual estimates of perceived size and distance. For every combination of size and distance, Rubik's cubes were perceived as larger and farther than the dice, even during binocular viewing at near distances (<1 meter), when oculomotor cues are particularly strong. For size perception but not distance perception, the familiar size effect was significantly stronger under monocular pinhole viewing than binocular viewing. These results suggest that (1) familiar size affects the accuracy of perception, not just the speed; (2) the effect occurs even when oculomotor cues are available; and (3) size and distance perception are not perfectly yoked.

## Introduction

The visual system must infer the properties of objects and scenes in the real world from the images that are projected on the retinas. For example, accurate visual estimates of physical size and distance are essential for visually guided actions, such as grasping (Jeannerod, 1981), and may also facilitate object recognition (e.g. Holler, Behrmann, & Snow, 2019).

Based on viewing geometry, there is a direct relationship between the physical size and physical distance of a real-world object and the retinal angle that it subtends on the two retinas. Observers’ estimates of size and distance can be affected by retinal size; specifically, retinally larger objects are perceived as being closer than retinally smaller objects (Sousa, Brenner, & Smeets, 2011; Sousa, Smeets, & Brenner, 2012). However, retinal angle on its own is insufficient to infer physical size and distance correctly because many combinations of physical size and distance can yield equivalent retinal angles. As such, retinal size must be combined with depth information to infer distance, which can then be used to infer size. This depth information can be provided by vergence (the angle between the two eyes as they fixate on an object) and accommodation (the change in focus of the eyes’ lenses), accompanied by changes in pupil size (the amount of light entering the eyes) in the “near triad” of oculomotor factors. Vertical disparities may also provide an absolute cue to distance (Brenner, Smeets, & Landy, 2001; Rogers & Bradshaw, 1993), but their utility appears limited to very large surfaces (20 degrees of visual angle; Bradshaw, Glennerster, & Rogers, 1996; Rogers & Bradshaw, 1995). A classic study by Holway and Boring (1941), found that observers scaled their estimates of size for a neutral object based on distance (size constancy) even when retinal angle remained constant if oculomotor depth cues were available. Nevertheless, the importance of oculomotor cues in estimating distance (and thus size) has long been debated (e.g. Fisher & Ciuffreda, 1988; Hastorf & Way, 1952; Holway & Boring, 1941).

Familiar objects also provide another strong cue – familiar size (Berkeley, 1709). Even brief hands-on experience with a real object can enable adults (Marotta & Goodale, 2001) and infants (Granrud, Haake, & Yonas, 1985) to learn the typical size of the object. This learned information can then be used to infer the expected size of the object, the distance that would be consistent with the expected size, and the expected size of other objects in the scene. Indeed, people's estimates of size from memory with the eyes closed are indistinguishable from their estimates of size during direct viewing (Bolles & Bailey, 1956; Churchill, 1962). Familiar size is particularly useful for perceiving images, which are often presented with erroneous relationships among distance (and thus vergence/accommodation), size, and retinal angle. As such, familiar objects (such as a person or a common object like a coin) are often placed in photographs to provide a size reference.

Evidence suggests that the speed of object perception is affected by familiar size even under binocular viewing when full cues to size and distance are available. For example, when participants judged the relative physical sizes of images of familiar objects on a computer screen, judgments were faster for object pairs in which relative sizes were congruent with familiar sizes (e.g. a large horse and a small clock) than pairs with incongruent familiar sizes (e.g. a small horse and a large clock; Konkle & Oliva, 2012). Thus, it appears that familiar size may be processed automatically. However, this study measured the speed of size perception rather than the accuracy.

One interesting question is how much familiar size affects the accuracy of perceived size (and perceived distance, which may be expected to be yoked) in the real world, when vergence and accommodation signals are available and valid. In such cases, does the visual system rely solely on oculomotor cues or is familiar size also factored in?

Historically, researchers examining whether familiar size affects accuracy of size and distance perception have found contradictory results. Under scrutiny, it becomes evident that these research groups approached their research questions from two different philosophical perspectives, which led to different methodologies and thus different findings.

One line of researchers, termed the “empiricists” or “transactionalists”, postulated that the visual system “takes into account” learned object features, including familiar size together with oculomotor cues (Ittelson & Ames, 1950; Kilpatrick & Ittelson, 1953). Presumably, along with object familiar size, the relationship between retinal angles and physical distances of familiar objects is also learned. As such, object distance can be inferred from the combination of an object's familiar size and the retinal angle it subtends (an idea sometimes called the size-distance invariance hypothesis; Epstein, Park, & Casey, 1961; Kilpatrick & Ittelson, 1953).

Empiricists typically tested whether familiar size alone would be sufficient to compute physical distance in the absence of oculomotor cues. To do so, they strived to eliminate most binocular and monocular cues except familiar size and retinal angle by showing familiar objects (usually flat images like playing cards) in a dark tunnel or via mirrors, viewed monocularly (to eliminate vergence as a cue). Across many studies under these circumstances, atypically sized objects were perceived as being of typical size at distances that would be consistent with the retinal angle (Fitzpatrick, Pasnak, & Tyer, 1982; Gogel & Mertens, 1967; Higashiyama, 1984; Ittelson, 1951; Ittelson & Ames, 1950). For instance, in the absence of oculomotor cues, a person viewing a playing card that subtends 2 degrees × 2.8 degrees would perceive the card as being the standard size (i.e. 6.5 cm × 9 cm) at a the geometrically appropriate distance (186 cm), when in fact the actual size was atypical (e.g. double size) with the viewing distance adjusted accordingly (e.g. double distance; Epstein et al., 1961; Kilpatrick & Ittelson, 1953). Even those who measured both perceived size and perceived distance often used flat stimuli of questionable realism (e.g. projected transparencies of stamps or catalogs, which may have appeared simulated; Mershon & Gogel, 1975) or did not use familiar objects (Brenner & Van Damme, 1999). Although most studies found a familiar size effect, some studies did not, particularly when the object stimuli appeared obviously unrealistic, such that observers were less likely to assume that the stimuli were actually familiar objects (Gogel & Mertens, 1967). In sum, the empiricists typically found that the visual system relies on familiar size for distance perception even at near distances (0.5–2.3 meters), but they tested these effects only in the absence of vergence cues and using flat (2D) stimuli.

In contrast to the empiricists, another theoretical camp, the “nativists”, postulated that retinal projection in and of itself offers sufficient information about the geometry of the scene to infer distance, and thus size, such that inferences from learned experience are not necessary (Gibson, 1959; Gibson, 1972). In their experiments, the nativists investigated the perceived sizes of real wooden chairs manufactured at three sizes – typical, oversized or undersized – and placed them in a football field (Franklin & Erickson, 1969), on a rooftop (Predebon, Wenderoth, & Curthoys, 1974), on a dirt road (Fillenbaum, Schiffman, & Butcher, 1965), or in a park (Slack, 1956). In such natural scenarios, the full range of depth cues, including both pictorial and binocular/oculomotor cues, were available. Under these conditions, the nativists found a negligible effect of familiar size on size perception at distances less than 7 meters (Schiffman, 1967) and, in some but not all studies, even at farther distances (Fillenbaum et al., 1965). Specifically, atypically sized objects were correctly perceived as being different from the norm, supporting the nativists’ claims that environmental cues alone were sufficient and that familiar size was not an essential cue for size perception. Note, however, that under the natural viewing conditions that were used, all possible distance and size cues were present, including textures and relative familiar sizes of objects in the scene. Thus, one possible interpretation is that accurate size perception of the target object was cued by its relations with surrounding familiar objects. For example, the size of an atypical chair may have been perceived correctly in part because of its relative size compared with other familiar objects or textures (e.g. the blades of grass beside it; Franklin & Erickson, 1969). Importantly, at far distances (beyond 7 meters), familiar size began to affect perception (Franklin & Erickson, 1969; Predebon et al., 1974; Schiffman, 1967; Slack, 1956), possibly due to the reduced utility of oculomotor cues at greater distances (Kaufman et al., 2006).

Given substantial methodological differences between empiricists and nativists (in addition to differences in their philosophical perspectives), the lack of consensus regarding the degree to which participants rely on oculomotor versus familiar-size cues comes as no surprise. Key differences in their approaches include: (1) the dependent measures used (distance perception *vs.* size perception), (2) the nature of the stimuli (2D images *vs.* real 3D objects), and (3) the absence versus availability of depth cues other than familiar size (specifically, oculomotor and pictorial cues). Any of these three factors could account for conflicting findings.

The aim of the present study was to measure the perceptual accuracy of (1) both size and distance estimations (2) for genuinely real three-dimensional objects, which are most relevant for understanding natural vision (3) with and without oculomotor cues at close viewing distances where oculomotor cues are strongly informative (Foley, 1980; Wallach, Gillam, & Cardillo, 1979).

**Figure 1.**
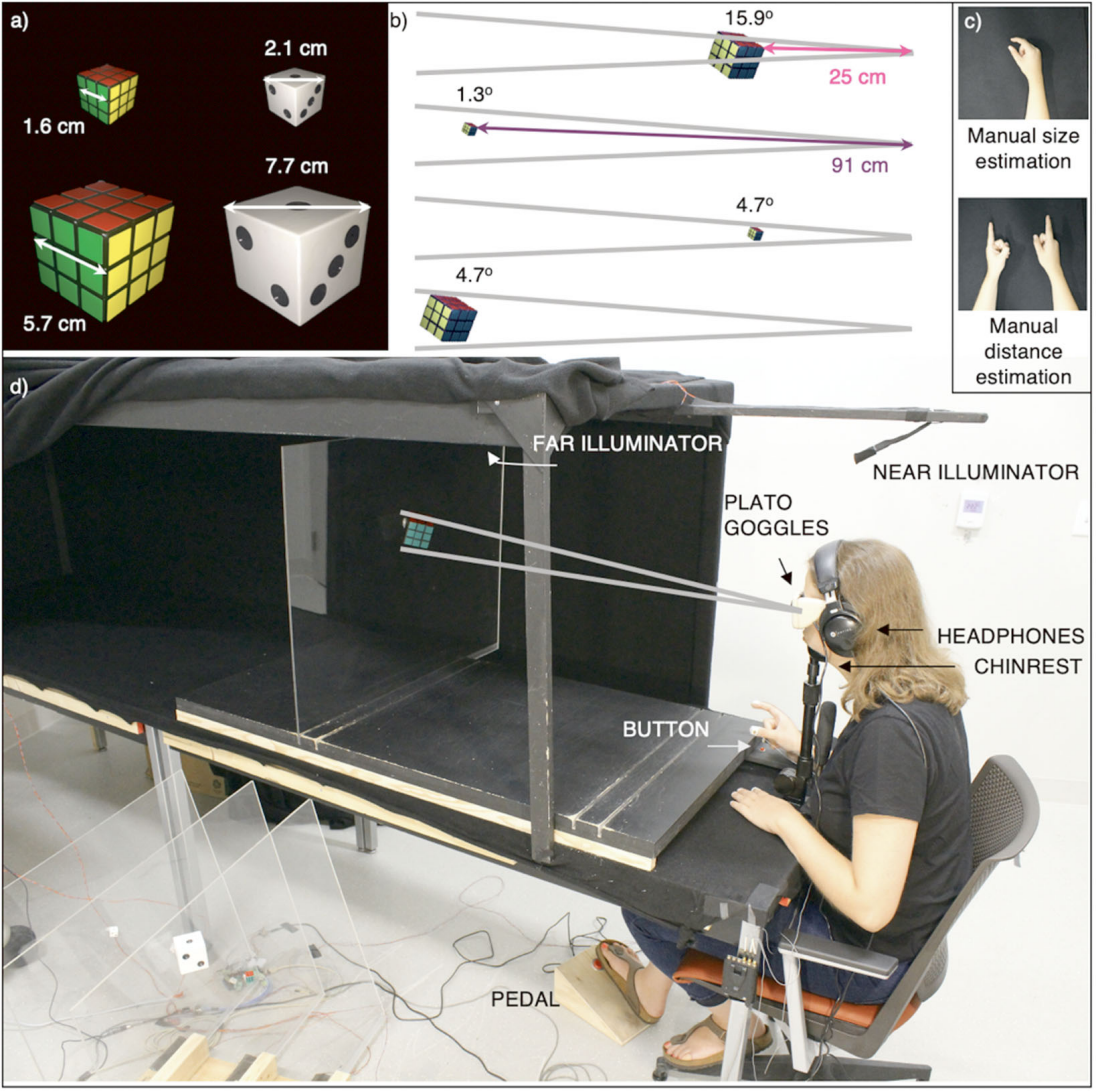
**Experimental Set-up.** (**a**) Object stimuli as seen from the participant's viewpoint. Rubik's cubes and dice were manufactured in two sizes. Objects were viewed from the perspective shown, such that three sides of each cube were visible, and the object appeared bilaterally symmetrical. Each object was presented in isolation in the absence of any pictorial cues, even shadows, and appeared to be floating in front of blackness. Physical sizes of the objects were calculated based on the width of the cube on each side, but retinal angles are described in terms of the size and distance of the widest part of the object as it would appear on the retina. (**b**) The combinations of two sizes and two distances yielded three possible retinal angles. Viewing distances were calculated as the distance from the participants’ eyes to the nearest tip of the tilted object. Note that two combinations (small near and large far objects) yielded identical retinal angles (4.7 degrees). (**c**) Participants indicated perceived sizes and distances using a manual estimation. They estimated perceived size by adjusting the distance between the thumb and the index finger on the right hand. They estimated perceived distance by adjusting the distance between the two index fingers. The order of reporting (size before distance or distance before size) was randomized and counterbalanced. (**d**) Experimental setup. Participants viewed objects in a tunnel lined with black cloth. Objects were mounted on transparent Plexiglas sheets that slid into grooves in the wooden base of the tunnel to ensure placement at the correct distance. Participants were seated and used a chinrest to ensure distances were fixed and to reduce contributions to distance perception from motion parallax cues. Prior to the start of each trial, participants began with the fingers of the right hand pinched together to press a button, and LCD (PLATO) goggles remained opaque. At the start of the trial, an audio instruction specified which of the two percepts (size or distance) to report first and a spotlight at the appropriate distance illuminated the object. Participants could view the objects for as long as they wanted, but as soon as the hand lifted off the button, the goggles became opaque such that manual estimations were performed without visual feedback. Once satisfied with their manual estimation, participants pressed the pedal with their right foot to record the position of their digits using a motion capture system (not shown). For illustrative purposes, the room is lit, the lateral side of the visual tunnel appears open, and other stimuli are visible. However, the actual experiment took place in the dark room, with cloth draped over all sides and with only one stimulus in view at any time, and with the stimuli hidden from sight prior to the start of the experiment.

We had participants manually estimate the size and distance of real three-dimensional (3D) objects with identical volumetric shapes (cubes) but different familiar sizes (i.e. Rubik's cube versus playing die). The chosen objects had strict canonical sizes to evoke more consistent memories of familiar sizes than objects with variable physical sizes (e.g. books; Haber & Levin, 2001). The two objects were manufactured at two physical sizes corresponding to their own familiar sizes (5.7-cm Rubik's cube and 1.6-cm die) and each other's familiar sizes (1.6-cm Rubik's cube and 5.7-cm die; Figure 1a). We were limited to two objects by the constraints of manufacturing realistic objects and the rate at which real stimuli can be presented; nevertheless, with two stimuli, our experiment had double the number of familiar objects tested in most previous experiments (e.g. Epstein et al., 1961; Franklin & Erickson, 1969).

The objects were placed at two possible distances, 25 cm and 91 cm, both of which were very near to the participant to enable potent oculomotor cues to distance (Wallach et al., 1979). These distances were much closer than previous studies, which ranged from 7 to 40 meters (Fillenbaum et al., 1965; Franklin & Erickson, 1969; Predebon et al., 1974; Slack, 1956). Vergence angle decays exponentially with distance and, accordingly, vergence is weighted more heavily in distance estimation at close distances (Tresilian, Mon-Williams, & Kelly, 1999). Thus, by using close distances (<1 meter), our design enables us to perform a strong test of the nativists’ hypothesis that oculomotor cues can be used to perceive distance accurately, and thus to infer size accurately. The combinations of physical sizes and distances were carefully chosen such that two combinations (near small and far large) subtended identical retinal angles.

We contrasted size and distance perception both with full oculomotor cues (binocular viewing) and without (monocular viewing through a pinhole to eliminate vergence and stereopsis, and to minimize accommodation cues). We presented the objects in isolation, rather than in the context of other objects, such that the only familiar size cue present in the scene was offered by the target stimulus (but not its relationship to other elements in the scene).

If familiar size affects object perception, we expected that the Rubik's cubes would be perceived as larger and farther than dice, even when the objects’ physical sizes and distances were matched. We term this difference the familiar size effect (FSE). Although familiar size would be expected to affect object perception during monocular pinhole viewing due to the lack of other depth cues (Fitzpatrick et al., 1982; Gogel & Mertens, 1967; Higashiyama, 1984; Ittelson, 1951; Ittelson & Ames, 1950), predictions are less certain for binocular viewing. On the one hand, if the nativist viewpoint is correct that the visual system bases perception solely on scene geometry, then familiar size should not affect perception when full oculomotor cues are available. On the other hand, if the empiricist viewpoint is correct and the visual system relies on learned object properties, then familiar size may indeed affect object perception even in the presence of full oculomotor cues.

## Methods

### Participants

Data from 32 participants (19 women and 13 men, age range = 17–22) were analyzed. Participants were recruited from the undergraduate psychology research participant pool at the University of Western Ontario and through recruitment posters placed on campus. They were all right-handed, as assessed by a scale from an adapted Edinburgh Handedness Inventory (EHI; Oldfield, 1971), performing 96% of tasks listed with the right hand (SD = 0.5%). All participants had normal stereoscopic acuity (*M* = 79 arcsec, SD = 77 arcsec), as assessed by the TNO stereo-test (Lameris Ootech, United Kingdom), and had normal or corrected-to-normal vision with no history of strabismus. All participants reported that they were familiar with a typical Rubik's cube and a typical die prior to the study but, importantly, were naïve with respect to the possibility of unusually sized objects in the study. They were also able to accurately draw squares representing the sizes of these familiar objects relying on their memory. The drawings of a Rubik's cube and a die differed by *M* = 4.4 cm (SD = 0.42 cm), matching closely the actual size difference between these objects in the real world (4.1 cm), indicating an accurate memory of their familiar sizes. At the start of the session, participants signed informed consent forms that were approved by the Non-Medical Research Ethics Board of the University of Western Ontario, in accordance with the 1964 Declaration of Helsinki. Participants were either compensated financially or given a course credit for their participation.

In addition to the 32 participants whose data were analyzed, partial data were collected from 23 additional participants, including 11 pilot participants. In these cases, full data sets were discarded if data from both trials for any one condition were missing due to occlusion of markers detected by a motion capture system. For the initial participants, the full extent data loss was only realized in postprocessing; this led to changes in data collection to minimize data loss in the later participants (including repositioning of the motion capture cameras and new code to enable the experimenter to check data quality on the fly and re-collect missing trials).

### General procedure

Apparatus and procedures are shown in Figure 1a to d. By design, the combinations of physical sizes (and distances) yielded an emergent variable of retinal angle with three levels: 1.3 degrees, 4.7 degrees, and 15.9 degrees (see Figure 1b), where the retinal angle refers to the widest projection on the retina (see Figure 1a). That is, two combinations of size and distance (1.6-cm objects viewed at 25 cm and 5.7-cm objects viewed at 91 cm) yielded a matched retinal angle of 4.7 degrees. Other combinations yielded retinal angles of 1.3 degrees (1.6-cm objects at 91 cm) or 15.9 degrees (5.7-cm objects at 25 cm).

The dependent variables were perceptual estimations of size and distance of objects. Immediately after seeing an object, participants moved their fingers apart to show perceived sizes and distance (i.e. manual estimation task; see Figure 1c) without visual feedback of either objects or hands (i.e. an open-loop estimate).

To eliminate other pictorial cues, objects were mounted on transparent acrylic sheets and presented one at a time in a tunnel in a dark room (akin to Ittelson & Ames, 1950, who presented 2D stimuli in similar viewing conditions), appearing to float freely in front of the dark background without visible shadows or background textures (see Figure 1a).

#### Pre-test

##### Apparatus and calibration of line of sight

As shown in Figure 1d, participants looked down a long custom-made tunnel (335 cm deep × 76 cm wide × 73 cm high), comprised of a wooden frame painted flat black and draped with black fleece. A standard chinrest was used in order to direct each participant's line of sight (i.e. Cyclopean view) horizontally toward the protruding vertex of each cubic object (located 40 cm above the table). To ensure this critical alignment, a simple task was devised wherein each participant raised or lowered the chinrest until two circles forming a bull's eye appeared aligned (that is, when the outer diameter of a closer cardboard circle appeared nested within the inner radius of a further cardboard donut). This alignment was performed by alternately opening one eye at a time, until the view with both eyes was symmetrical, thus, binocular disparity between a close and a far circle, if viewed binocularly, did not affect the accuracy of the visual alignment with the line of sight. For the monocular pinhole viewing condition, participants wore commercially available plastic pinhole glasses with a clean-cut 1-mm pinhole along the line of sight of their dominant eye (and all other pinholes were closed), and with the nondominant eye completely covered.

##### Motion capture

To accurately measure manual estimates of perceived size and distance, three infrared-emitting diodes (IREDs; Optotrak; Northern Digital, Waterloo, Canada) were secured on the participants’ right thumb tip (distal phalanx), and both right and left index fingertips (distal phalanxes), close to the fingernails. Participants were encouraged to assume a natural hand position (matching the natural kinematics of the hand; see Figure 1c) for the manual estimates, without rotations that could block the cameras’ views. Two three-camera opto-electronic recording systems (Optotrak 3020; Northern Digital) were positioned approximately 2.5 meters above floor level and laterally to each side of the tunnel, pointed directly toward the table surface, above which the manual estimations were made. As such, participants were instructed to orient their hands in the anatomically neutral position (thumb side up; see Figure 1c), such that when the thumb and finger (for size estimation) and both index fingers (for distance estimation) were brought together in a “pinching” action, these IREDs were adjacent to one another and faced upward.

##### Calibration of manual estimates

Because manual estimates of size and distance should be based on the distance between the digit pads, but IREDs were placed next to the digit nails (to prevent IRED occlusion during motion capture), the distance between the IREDs when fingers were brought together was subtracted from the perceptual size estimates. In addition, to capture any potential shift of the IREDs throughout the duration of the experiment, calibration trials were performed before and after the main task to determine the difference between the IRED positions of the fingers being brought together before and after the main trials; reassuringly, this shift was negligible, less than one millimeter (*M* = 0.99 mm, SD = 1.08 mm).

##### Experimental testing

To eliminate pictorial cues except familiar size, room lights were turned off throughout the study. A headlamp and a set of light-emitting diodes (LEDs) aided the experimenter in presenting the objects specified in predetermined orders. To eliminate motion parallax as a depth cue, participants placed their heads on a chinrest. To restrict their vision in between the trials, participants wore liquid-crystal display (LCD) Portable Liquid-Crystal Apparatus for Tachistoscopic Occlusion (PLATO) goggles (Translucent Technologies Inc.) for which the lenses could be rendered transparent or opaque under computer control. To obstruct potential auditory cues to distance while the objects were placed by the experimenter, participants wore over-the-ears, noise-cancelling headphones that played white noise between the trials.

##### Task

Once the object was placed in the proper location and the participant pressed a button with the right index finger and thumb pinched together, a computerized female voice instructed participants to start the trial and indicated which perceptual measure was to be estimated first (i.e. “size, then distance” or “distance, then size”). After a random delay of 250 to 1000 ms after the end of the instruction, the goggles became translucent and one of two white LEDs (near or far illuminators in Figure 1d) illuminated the object from above.

Participants were instructed to indicate the perceived size of a single side of each cubic object by adjusting the aperture between the right index finger and thumb as if they were to grasp it (objective instruction; Predebon, 1992). They were instructed to indicate the perceived distance of the object by adjusting the distance between the right and left index fingers to match the distance from their face to the front vertex of the object (along a left-right axis perpendicular to the actual distance). Participants were instructed to “glance at the object” and “base estimations on first impressions.” As soon as participants lifted their fingers off the button to begin manual estimation, the goggles closed, eliminating visual feedback. On average, participants allowed themselves *M* = 2.33 seconds (SD = 0.83 seconds) to view an object. Participants had up to 10 seconds to make estimations of both size and distance; on average, participants took *M* = 2.85 seconds (SD = 0.65 seconds) to perform each estimation. To enter their response, they pressed a foot pedal after each estimation. Size and distance estimates were measured at the moment (i.e. a single frame of the Optotrak data stream) of the foot pedal press initiated by the participants when they were satisfied with their estimates.

##### Trials

Participants first performed a sufficient number of practice trials to ensure that the task had been mastered under the experimental viewing conditions. Practice trials under relevant viewing conditions were performed directly prior to each viewing condition. For the practice trials, novel, unfamiliar cubes (made of light blue extruded polystyrene) were utilized; the practice objects came in a range of sizes appearing at a range of distances that differed from those of the experimental objects. The experimental objects were first viewed only for the main trials (special care was taken to hide experimental objects out of view before showing the first experimental object inside the visual tunnel). The main trials were ordered in a mixed design. Viewing condition was blocked within participants and counterbalanced between participants (i.e. monocular pinhole viewing preceded binocular viewing or vice versa). Physical distance of the objects was blocked within the viewing condition block and, also, counterbalanced between participants (i.e. near distance preceding far distance or vice versa). The choice to block physical distance was made to facilitate comparisons with a parallel functional magnetic resonance imaging (fMRI) study (which required distance blocking to avoid frequent changes in vergence, which can evoke large changes in brain activation; Quinlan & Culham, 2007). Object physical sizes (small versus near) and IDs (die versus Rubik's cube) were quasi-randomized within participants and counterbalanced between participants. Specifically, objects with the same physical sizes but different identities occurred in temporal proximity to each other, to ensure that comparison of perceptual estimations for objects with identical sizes and distances but different IDs/familiar sizes would be minimally affected by the change in the actual physical dimensions of objects. For each trial, the size and distance estimations were performed sequentially, with the order of the type of estimations (i.e. size, and then distance, or vice versa) counterbalanced between the trials and between participants. Therefore, there were two trials for each of the 16 conditions, for a total of 32 trials. Each object was presented by sliding an acrylic sheet, with the object mounted in the center, into a slot in the bottom of the tunnel. Despite the differences in the physical sizes of the objects, both the height in the visual field and the viewing distance (for far and near, respectively) of the protruding front corner of every object were matched between the objects and the participants. Manual changes of objects between trials resulted in approximately 30-second intertrial intervals (ITIs) and this ITI was used even when the same condition occurred two times in a row. Moreover, to keep the distance at which the object was placed unknown to the participants before the opening of the goggles, in addition to the white noise played in the headphones during the ITIs, the experimenter slid another acrylic sheet at a different distance in and out of the tunnel on every trial, ambiguating cues to auditory localization. The entire session, including consent, setup, and data collection, took approximately 1.5 hours.

##### Error feedback

After each trial, participants received auditory feedback indicating whether they completed the task within the time limit (10 seconds). Trials that exceeded the limit were repeated immediately. On average, *M* = 0.75 (SD = 1.32) trials per participant were repeated due to the timing issues.

Several trials in the early participants had IREDs blocked at the critical moments when the foot pedal was pressed. When the problem was discovered in data processing, additional checks and feedback were introduced: after trials where the relevant IREDs were not tracked during each pedal press, a series of tones was played, and the trial was repeated immediately. On average, *M* = 3.2 (SD = 4.72) trials were repeated due to tracking issues.

## Materials

As die-sized Rubik's cubes and Rubik's cube-sized dice do not exist for commercial purchase, we custom made highly realistic Rubik's cubes and dice at two different physical sizes (1.6 and 5.7 cm) representing the familiar and reversed sizes. First, Rubik's cubes and dice at each size were machined from hardwood (i.e. maple), complete with their respective grooves and dimples at the appropriately scaled size and depth. These objects were finely sanded until smooth and then sealed with liquid polyurethane until the surface texture matched that of smooth plastic (i.e. matching the materials typically used to manufacture Rubik's cubes and dice). Silicone rubber molds (Mold Max 20; Smooth-On, Inc., Macungie, PA, USA) were then created so that perfect replica stimuli could be made from a more durable plastic. Once the silicone had cured and the molds were demolded, a release agent was sprayed inside the molds prior to casting. To reproduce the original models, a white or black liquid resin (AlumiRes RC-3; Allumilite) was poured into the prepared molds and left to cure, thus creating the final stimuli objects. To reproduce the Rubik's cube colorations, custom-cut vinyl stickers (red, green, and yellow) were placed on the large and small Rubik's cube faces, with all proportions maintained across both physical sizes. Each of the four objects was mounted on a short acrylic rod and secured with a hot glue close to the center of a transparent acrylic sheet (61 cm high and 76 cm wide; see Figure 1d), such that the front protruding corner was centered left-to-right and was at 40 cm from the tabletop. The protruding corner was angled such that participants could see three faces of the cubes, which appeared bilaterally symmetric and three-dimensional. Under the experimental lighting conditions, the surface of the supporting acrylic sheets was not visible to participants (as experimenters were careful not to create fingerprints by wearing gloves during the trials and wiping the surfaces prior to each session) and no shadows were visible. As such, anecdotally, the objects appeared to be floating in space without any pictorial cues to their relative sizes and/or distances. The acrylic sheets with the objects mounted on them were placed in one of four groves, which were cut in the base of the tunnel (two for the near distance and two for the far distance), such that the nearest corner of each cube appeared at one of two distances (25 or 91 cm). The objects were illuminated by one of two white LEDs at either the near or far distance. Both near and far LEDs were equidistant from the objects at the respective locations. Illumination was carefully matched by the experimenter by simultaneously inserting near small and far large objects (viewed slightly off the line of sight to avoid occlusion by the closer object) with the same identity and adjusting the intensity of each LED until subjective equiluminance was achieved. Even though objects were not presented further than 91 cm, a long tunnel was used so that participants did not expect object distances to fall within a narrow range. Specifically, the length of the tunnel (335 cm) was chosen to match the distance at which a real-world-sized Rubik's cube would have to be placed to appear to subtend 1.3 degrees of a retinal angle.

All computers ran Windows OS (Microsoft Inc.). The Optotrak system was controlled using in-house software (OTCollect programmed by Haitao Yang) and sampled at 100 Hz. MATLAB (2013; The MathWorks Inc., Natick, MA, USA) was used to present audio (instructions, white noise, and feedback tones) and interface with the input/output device (Arduino Mega 2560 Rev3) to control the PLATO goggles, hand release button, foot pedal, and LEDs.

## Results

**Figure 2.**
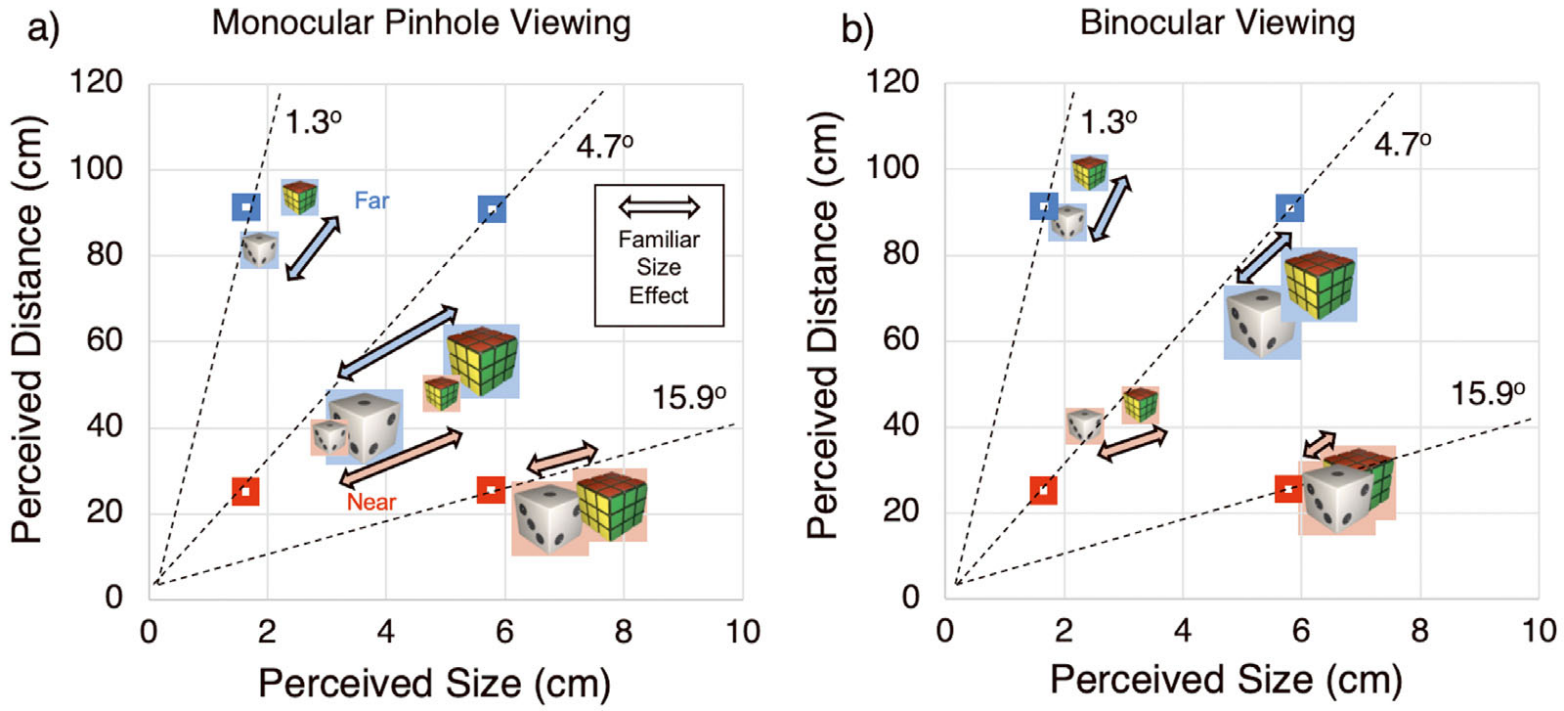
**Perceived size and distance for all combinations of object identities, sizes, distances,and viewing conditions under monocular pinhole viewing** (**a**) **and binocular viewing** (**b**) **conditions.** The center of the colored icons represent the average perceived size on the x-axis and average perceived distance on the y-axis for the large and small Rubik's cubes and dice (as indicated by icon size) at near (red) and far (blue) distances. The hollow squares indicate four actual combinations of physical sizes and distances. Oblique dashed lines represent the lines of constant retinal angles, such that objects of a given size and distance, falling along the dashed lines, would subtend the same retinal angles (e.g. 1.6-cm objects at 25 cm and 5.7-cm objects at 91 cm, both subtend 4.7 degrees retinal angle). Arrows indicate the familiar size effect (FSE), that is, the difference in perceived size and distance between the Rubik's cube and die when presented at the same physical size and distance. Notably, the strongest FSEs were observed during monocular pinhole viewing for the objects with intermediate retinal angle (4.7 degrees).

We collected perceptual estimates of both size and distance. As this study involved a new paradigm that will provide behavioral data for a similar neuroimaging paradigm and for future studies in virtual reality, we first qualitatively examined perceived size and distance together to gain insights into the nature of familiar size processing (Figure 2). After presenting the qualitative assessment, we describe the outcome of statistical tests to demonstrate that the described effects are statistically robust and reliable.

### Qualitative analysis of perceived size and distance

Figure 2 plots participants’ estimates of perceived size and perceived distance for every condition, as indicated by small and large icons of Rubik's cubes and dice, color coded to indicate presentation at near (red) or far (blue) distances. Figure 2 also shows objects’ veridical sizes and distances (hollow squares) and retinal angles subtended by the objects at a particular combination of size and distance (dashed black lines). FSEs are indicated by the arrows showing the difference in perception between the Rubik's cube and die at each combination of physical size and distance.

Several interesting effects can be noted in Figure 2.

First, familiar size affected size and distance estimations under binocular viewing, although the effect was stronger for monocular pinhole viewing. As indicated by the arrows depicting the magnitude of the FSE, for all viewing conditions, Rubik's cubes were perceived larger and farther than dice (that is, in Figure 2, the Rubik's cubes icons appear to the right and above of the dice icons). These results show that even when vergence and accommodation provide strong cues to depth at near distances (<1 meter), they reduce but do not eliminate the familiar size effect.

Second, familiar size had a particularly strong effect on size perception for the intermediate retinal angles (4.7 degrees) compared to the more extreme retinal angles (small far 1.3 degrees and large near 15.9 degrees items). Under both viewing conditions, the perceived sizes and perceived distances were in the vicinity of the veridical ones (i.e. see in Figure 2, object icons cluster around the hollow squares), especially for extreme visual angles. The FSEs were especially strong under monocular pinhole viewing for the objects with the intermediate retinal angles (4.7 degrees). Specifically, under monocular pinhole viewing, the 4.7 degrees objects were perceived according to their IDs/familiar sizes (e.g. see in Figure 2a, the large far die is perceived as most similar to the small near die); whereas under binocular viewing, the objects were perceived according to their physical sizes and physical distances (e.g. see in Figure 2b, the large far die is perceived as most similar to the large far Rubik's cube).

Finally, Figure 2 also shows that, as would be expected (Gilinsky, 1951), perceived size and distance co-varied, albeit imperfectly. That is, the combination of perceived size and distance falls close to the lines of constant retinal angle (see dashed lines in Figure 2) that would be expected from size constancy (Gilinsky, 1951; Sperandio & Chouinard, 2015). For example, when the monocularly viewed large (5.7 cm) far (91 cm) die (with an actual retinal angle of 4.7 degrees) is perceived smaller than it really is (3.6 cm), it is also perceived as closer (39 cm), a combination that would lead to a retinal angle (5.3 degrees) similar to the actual one. Notably, however, this relationship is imperfect, with perceived distance being closer than what would be required (given the perceived size) to preserve complete size constancy.

### Statistical analysis of perceived size and distance

The statistical reliability of these qualitative observations was examined with two analyses of variance (ANOVAs) for the dependent variables of perceived size and perceived distance. Because the two ANOVAs involved 30 statistical tests (2 ANOVAs with 4 main effects, 6 two-way interactions, 4 three-way interactions, and 1 four-way interaction), to limit the likelihood of finding any significant effect due to chance to less than 5% (Cramer, van Ravenzwaaij, Matzke, Steingroever, Wetzels, Grasman, Waldorp, & Wagenmakers, 2016), we only considered effects with *p* < 0.0016 = 0.05/30 (consistent with a Bonferroni correction). Interactions that reached statistical significance were dissected with two-tailed paired-samples post hoc *t-*tests. Because stringent correction of the ANOVAs limited the likelihood of finding interactions due to chance (to 0.05 across all tests), we did not apply a correction for multiple comparisons on the post hoc *t-*tests (Rosenthal & Rosnow, 1991, p. 328). To address concerns raised by a reviewer, we also investigated potential differences in reaction time and estimation time between the two objects; in this case, we did not correct for multiple comparisons because we wanted to show that there were no meaningful differences even under relatively liberal statistical thresholds. Effect sizes were quantified using partial eta squared (*η_p_*^2^). Error bars on graphs represent 95% confidence intervals (CIs). Statistical analyses were conducted with Jamovi (The jamovi project (2021). *Jamovi* (Version 1.6) [Computer software]. Retrieved from https://www.jamovi.org) statistical software.

**Figure 3.**
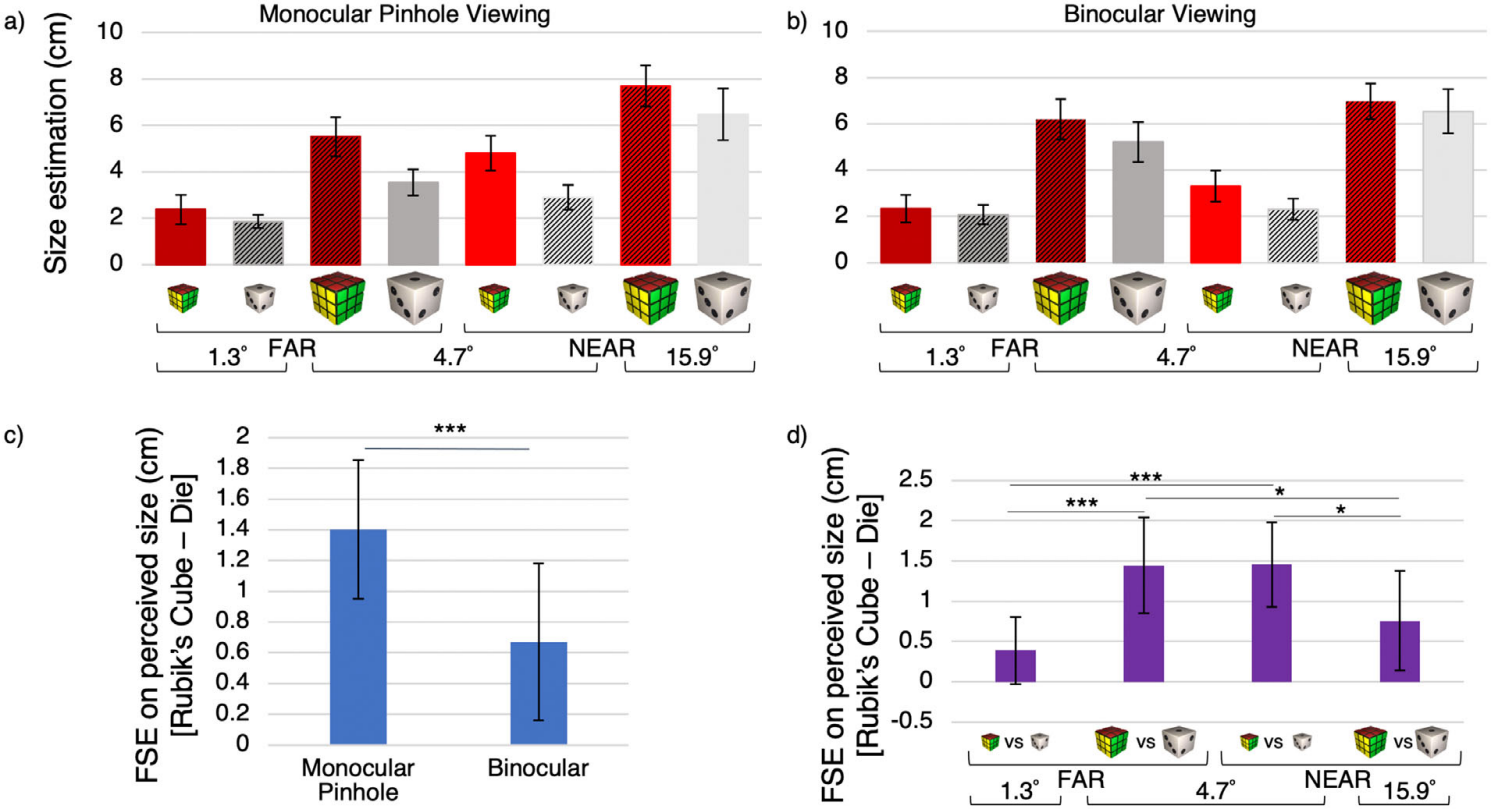
**Familiar size effect (FSE) for perceived size.** The top row shows participant mean size estimates for each combination of ID, physical size, and physical distance in the monocular pinhole (**a**) and binocular viewing conditions (**b**). Rubik's cubes (red bars) were perceived as larger than dice (gray bars), even when their physical sizes and distances were matched. Grated and plain bars show objects with familiar sizes congruent and incongruent with their physical sizes, respectively. To examine simple main effects of ID and its interaction with viewing condition, (**c**) shows the FSE = perceived size for (Rubik's cube – die) separately for monocular pinhole and binocular viewing. To better understand the statistical interaction of ID, size, and distance (**d**) shows the FSE for the four combinations of size and distance (collapsed across viewing condition because there was no four-way interaction). FSEs were greater in conditions with objects that subtended retinal angles of 4.7 degrees, which constituted visually ambiguous conditions with objects with different physical sizes and distances subtending matched retinal angles. Error bars indicate 95% confidence intervals (CIs). *Note* ***p < 0.001, **p < 0.01, *p < 0.05.

**Figure 4.**
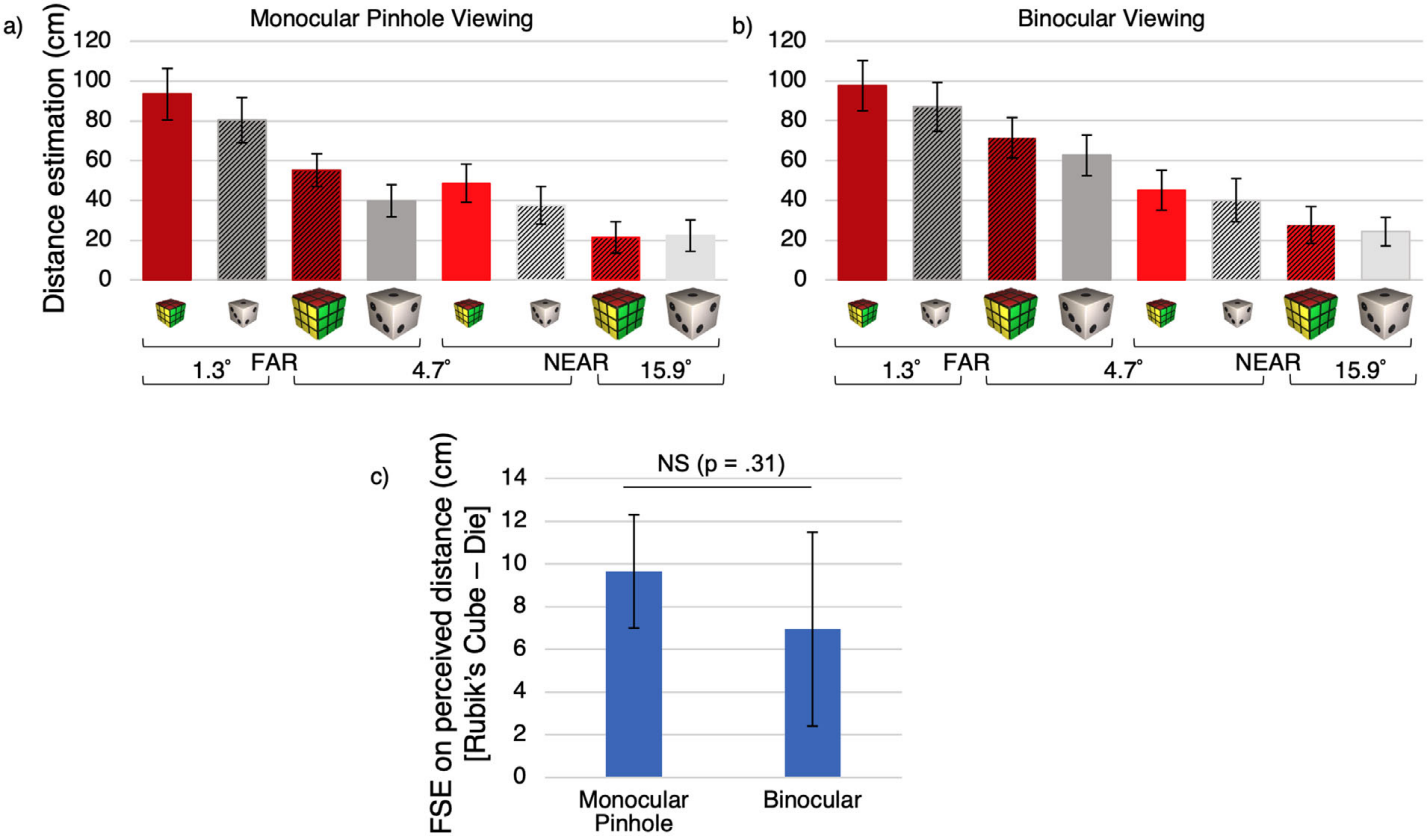
**Familiar size effect (FSE) for perceived distance.** The top row shows participant mean distance estimates for each combination of ID, physical size, and physical distance in the monocular pinhole (**a**) and binocular viewing conditions (**b**). Rubik's cubes (red bars) were perceived as farther than dice (gray bars), even when their physical sizes and distances were matched. Grated and plain bars show objects with familiar sizes congruent and incongruent with their physical sizes, respectively. As shown in (**c**), there was a significant FSE under binocular as well as monocular pinhole viewing. However, there was no significant difference in the magnitude of the FSE between the two viewing conditions.

For readers who wish to examine the data, we provide graphs for size and distance estimates and effects of greatest theoretical interest (Figures 3, 4). The ANOVAs also revealed other interactions of lesser interest, which can be inspected through additional resources on a data sharing repository (https://dx.doi.org/10.17605/OSF.IO/D5U62).

### Perceived size

Figures 3a and b depict perceived sizes of objects in all 16 conditions. We conducted a two IDs (Rubik's cube versus die) × two viewing conditions (monocular pinhole versus binocular) × two physical sizes (small versus large) × two physical distances (near versus far) repeated-measures ANOVA. Table 1 shows significant main effects and interactions. The independent variables interacted in multiple ways, including a three-way interaction of ID × physical size × physical distance.

**Table 1.** **Significant main effects and interactions of perceived size estimates**, as revealed by two IDs (Rubik's cube versus die) × two physical sizes (small versus large) × two physical distances (near versus far) × two viewing conditions (monocular pinhole versus binocular) repeated-measures ANOVA.

Effect	*F*(1, 31)	Effect size *(η*^2^)	*p* value
ID	23.5	.43	<0.001
Physical size	158.8	.84	<0.001
Physical distance	69.6	.69	<0.001
ID × viewing condition	13.3	.30	<0.001
Viewing condition × physical size	13.8	.31	<0.001
Viewing condition × physical distance	16.9	.35	<0.001
ID times physical size × physical distance	20.8	.40	<0.001

To simplify the interpretation, we computed a familiar size effect as the difference between perceived size for the Rubik's cube and die when other variables (physical size, physical distance, and viewing condition were matched), as represented by the arrows in Figure 2. This simplification helps to interpret the interactions between ID and other variables.

#### Familiar size affects size perception even under binocular viewing but to a lesser degree than monocular viewing

As shown in Figure 3c, the FSE was significantly greater than zero under binocular viewing (*p* < 0.05) as well as monocular viewing (*p* < 0.001) even though the binocular FSE was significantly weaker than the monocular FSE (as indicated by the significant interaction between ID and viewing condition, which is statistically equivalent to a *t-*test on FSEs between the two viewing conditions).

#### Stronger FSEs for size for intermediate versus extreme retinal angles

To interpret the significant three-way interaction of ID, physical size, and physical distance on perceived size, we collapsed the data across viewing conditions and computed the FSE for size for each of the four combinations of physical size and physical distance, as shown in Figure 3d (and as indicated by the arrows in Figure 2). As evident in Figure 3d, the three-way interaction was driven by stronger FSEs for objects with intermediate retinal angles (4.7 degrees) than extreme ones (1.3 degrees and 15.9 degrees). Specifically, post hoc *t*-tests showed that FSEs for the objects with intermediate angles (near small and far large) were significantly larger than for the objects with extreme retinal angles (near large, *t*(31) = 2.24, *p* = 0.032, *t*(31) = 2.09, *p* = 0.045; far small, *t*(31) = 6.14, *p* < 0.001, *t*(31) = 3.82, *p* < 0.001).

### Perceived distance

We conducted a two ID (Rubik's cube versus die) × two viewing conditions (monocular pinhole versus binocular) × two physical sizes (small versus large) × two physical distances (near versus far) repeated-measures ANOVA on perceived distance. Table 2 shows significant main effects and interactions. Because there were interactions between viewing condition and other variables, Figures 4a and b show the data separately for the two viewing conditions.

**Table 2.** **Significant main effects and interactions of the perceived distance estimates**, as revealed by two IDs (Rubik's cube versus die) × two physical sizes (small versus large) × two physical distances (near versus far) × two viewing conditions (monocular pinhole versus binocular) repeated-measures ANOVA.

Effect	*F*(1, 31)	Effect size *(η*^2^)	*p* value
ID	43.0	0.58	<0.001
Physical size	107.5	0.78	<0.001
Physical distance	131.9	0.81	<0.001
ID × physical distance	18.6	0.38	<0.001
Physical distance × physical size	18.3	0.37	<0.001

#### Familiar size affects distance perception under binocular viewing to a similar degree as monocular viewing

As shown in Figure 4c, the FSE for perceived distance was significantly greater than zero under binocular viewing (*p* < 0.05) as well as monocular viewing (*p* < 0.001). However, there was no significant difference between the magnitude of the FSE in the two viewing conditions (as indicated by a significant main effect of ID, but no significant interaction of ID with viewing condition, *p* = 0.31). Thus, whereas binocular viewing significantly reduced the FSE for size perception, there was no significant difference between viewing conditions for distance perception.

#### Reaction times and estimation times

Although we were primarily interested in perceived size and perceived distance, we also examined reaction time (from the moment the goggles opened until participants lifted the hand(s) to begin manual estimation) and the time taken to do the manual estimation (from the time the hand lifted off until the foot pressed a pedal to lock in the response) for the size and distance perception tasks. These data were analyzed to address the concerns of a reviewer that differences in perceived size and distance between the Rubik's cube and die could be due to stimulus differences such as salience. If so, one would expect that the more attentionally salient stimulus would be processed faster.

Because participants executed size and distance estimation sequentially (in counterbalanced order), the two tasks had a common reaction time. There was no main effect of identity (*p* = 0.213), as reaction times were similar for the Rubik's cube (*M* = 2299 ms) and die (*M* = 2371 ms). Reaction times were faster for binocular conditions (*M* = 2117 ms) than monocular conditions (*M* = 2553 ms), as indicated by a significant main effect of viewing condition (*F*(1,30) = 18.2, *p* < 0.001). No other main effects were significant, nor were any interactions (all *p* > 0.10).

Object identity did not affect estimation time for size and distance perception. There was no significant main effect of object identity for size (*p* = 0.68), although there was a trend for a main effect of object identity for distance (*p* = 0.07). Estimation times for the Rubik's cube and die were similar for size (*M* = 2804 versus 2821 ms, respectively) and distance (*M* = 2921 versus 2856 ms, respectively). There were no interactions between object identity and other factors for either perceived size or perceived distance.

Taken together, the analysis of reaction times and estimation times for the size perception task showed no obvious differences between the two stimuli that would account for the differences in perception.

## Discussion

These results indicate that familiar size affected both perceived size and distance, in line with the empiricists’ theoretical position (Ittelson & Ames, 1950; Kilpatrick & Ittelson, 1953). That is, Rubik's cubes were perceived larger and farther than dice when their actual physical sizes and distances were matched, which we term FSEs on size perception and distance perception, respectively. Most notably, familiar size affected perception even when potent oculomotor cues were available at near distances (<1 meter).

Generally, familiar size had a greater effect on object perception under conditions of high uncertainty. For perceived size, the FSE was stronger during monocular pinhole viewing than binocular viewing (see Figure 3c). In addition, for perceived size, the FSE was stronger for the two conditions with intermediate retinal angles (4.7 degrees) than extreme retinal angles (1.3 degrees and 15.9 degrees; see Figure 3d). Thus, for our limited range of stimuli, extreme retinal angles enabled quite accurate perceptual estimates, whereas intermediate retinal angles evoked less veridical perception, even when combined with oculomotor cues. Interestingly, perceived size for intermediate (4.7 degrees) stimuli was largely based on familiar size during monocular pinhole viewing, when depth cues were minimal, but largely based on physical size during binocular viewing, when rich depth cues were available. Notably, however, even in the binocular condition, familiar size still affected size perception. Familiar size also affected perceived distance, although the magnitude of the FSE did not differ statistically between binocular and monocular pinhole viewing (see Figure 4c).

When size and distance are examined together, it becomes evident that perceived size and distance are yoked, albeit imperfectly, resulting in partial size constancy (e.g. when perceived size decreased, the perceived distance also decreased, such that the combination of perceived size and perceived distance falls along a diagonal consistent with a given retinal angle, as shown in Figure 2).

The current results not only help to reconcile the longstanding debate about FSE between the empiricists and the nativists, but they also extend the conclusions that can be drawn from behavioral studies. A growing number of recent behavioral studies have found advantages in processing speed (namely, reaction times) for objects with familiar sizes that are congruent (versus incongruent) with their relative (Konkle & Oliva, 2012) or physical sizes (Fisher & Sperandio, 2018) in the real world. The current results show that familiar size also affects perceptual accuracy and that these effects are surprisingly potent, being present even when oculomotor cues to distance are available (and could be combined with information about distance to infer size).

The current experimental design provides several additional advantages over past research. First, the multifactorial design used here allowed us to assess the impact of multiple visual features that naturally co-occur in the real world (familiar size, physical size, retinal size, and physical distance) to examine effects on both size and distance perception. As a result, we show that although these percepts are correlated, the FSE on size perception was more dependent upon the available depth cues than the FSE on distance perception. This finding is consistent with other research showing that perceived distance, size, and shape are not always interpreted to be consistent with one another (Brenner & van Damme, 1999). Second, unlike past experiments that examined perception of a single familiar object (e.g. playing cards or chairs) at typical and atypical sizes, here, the stimulus set consisted of two objects with carefully chosen identities. These objects have (1) strict canonical sizes, each eliciting a specific familiar size representation (i.e. drawings of objects from memory differed by less than 5 mm between participants) and (2) matched shapes (cubes) viewed under the same conditions (perspective and lighting). As such, the two object identities acted as each other's controls and yielded robust FSEs. Third, we used real objects rather than images, which may be important because real objects are expected to have congruent familiar sizes, whereas images are not. That is, we are often surprised and amused to see miniatures or jumbo versions of real stimuli; whereas, under- or oversized photographs are commonplace and unremarkable (although photographic objects are recognized faster when their presented size matches their familiar size; Fisher & Sperandio, 2018). Moreover, real-world objects always produce consistent cues to depth, where retinal angle and oculomotor cues directly correspond to object physical size and distance. Thus, the current findings of strong FSEs under binocular viewing are even more surprising. Fourth, our inclusion of two combinations of size and distance that yield images subtending an equivalent retinal angle (4.7 degrees) eliminates potential confounds of retinal angle in size estimation. Even for these ambiguous retinal angles, we find FSEs on size and distance, which provides a counterpoint to a recent claim that FSEs are absent when confounds of retinal size are removed (Mischenko, Negishi, Gorbunova, & Sawada, 2020). Finally, although the use of only two object identities may limit generalizability, the current design remains an enhancement of past studies that only considered one object (e.g. Epstein et al., 1961; Franklin & Erickson, 1969).

### Reconciling the debate between empiricists and nativists

These results elucidate potential reasons for conflicting findings between the empiricists and the nativists. Recall the key differences between the experimental approaches of the two camps: whereas the empiricists typically examined distance perception of 2D images in highly controlled visual environments with restricted monocular viewing and found strong FSEs, the nativists typically studied size perception of real objects in natural environments with binocular and environmental cues and found negligible FSEs. Thus, the different conclusions could result from differences in the dependent variable, the types of objects, or the presence of oculomotor and pictorial cues.

Based on our results, even when oculomotor cues were present, familiar size affected both size and distance perception; thus, neither the dependent variables nor the availability of oculomotor cues can fully account for the differences in past findings. Our results suggest that the FSE for size was weaker under binocular viewing, when vergence, accommodation, and stereopsis cues were available, than under monocular pinhole viewing; however, significant FSEs were observed under both viewing conditions. Moreover, the current study showed the FSE at an unprecedently close distance with binocular viewing. Previous studies that investigated FSE at close distances (approximately 2 meters; Gogel & Newton, 1969; Higashiyama, 1984; Ittelson, 1951; Ittelson & Ames, 1950) implemented only monocular viewing; whereas, those that investigated FSE under binocular viewing, presented objects only at relatively far distances (typically beyond 20 meters; Fillenbaum et al., 1965; Franklin & Erickson, 1969). Notably, the current results suggest that familiar size contributes to depth perception even at close distances (less than 1 meter) where binocular cues are highly effective (Foley, 1980; Wallach et al., 1979).

Another key difference between the empiricists and nativists may be the presence of a naturalistic context only in the nativists’ experiments, with object stimuli presented in rich real-world environments with other objects and textures (e.g. a chair in a field of grass; Franklin & Erickson, 1969). In the current study, akin to the empiricists, an environmental context was completely absent, with the objects presented in complete isolation. In addition, akin to the empiricists, we find strong FSEs, even when oculomotor cues could be expected to produce accurate size and distance perception. Intriguingly, although the nativists used their results to argue against a contribution of familiar size, perhaps their participants were better able to infer the true size of the atypically sized target object (e.g. the chair) based on a relative comparison with other familiar objects and textures in the scene (e.g. blades of grass) that were of typical size.

Unlike the empiricists and the nativists, who studied perceived size and distance (respectively) in isolation, our results can elucidate the relationship between the two percepts. Consistent with Haber and Levin (2001), our results show that size and distance perception may not rely equally on the same cues (as expected from size constancy). Specifically, the FSE for size perception was significantly stronger for monocular (versus binocular) viewing and for intermediate (versus extreme) visual angles but no such effects were found for distance perception.

### Limitations

#### Are FSEs specific to the stimuli used?

One potential criticism of the experimental design we used is that only two object identities were tested. The two object stimuli differ in low-level visual properties: overall luminance, luminance contrast, and color, with the die being brighter, higher contrast, and monochrome compared to the dimmer, lower contrast, and multicolored Rubik's cube. Moreover, the two stimuli differ in their surface textures and the number of elements they contain. That is, while the die is a single item with dimples representing low quantities (1, 2, or 3 dots per side); whereas the Rubik's cube comprises a grid of 27 sub-cubes (with 9 elements per side).

Although we acknowledge these potential differences, they are unlikely to explain the effects found here. First, data from a follow-up project using sports balls with small familiar sizes (baseball, golf ball, and pool ball) and large familiar sizes (basketball, soccer ball, and volleyball) presented in virtual reality finds that perceived size and distance are clearly affected by familiar size (Hussey, Babin, Wilcox, & Culham, 2021). Second, there were no clear differences between the Rubik's cube and die in reaction times or estimation times that would indicate potential differences in salience.

#### Can the results be explained by the absence of other visual cues?

In our experiment, objects were presented in isolation, excluding other cues to depth that would be present in real-world scenes. Notably, four types of cues were absent: the relative sizes of other objects in the scene, vertical disparities for large surfaces, pictorial cues to depth (e.g. shadow and perspective), and motion parallax.

Our choice to present objects in isolation was deliberate. Had we presented multiple objects in the scene – an off-sized target object among other objects with true familiar sizes – we would not have been able to assess the contribution of oculomotor cues versus familiar size (because the findings would be affected by conflicting familiar sizes). In addition, presenting stimuli in isolation enabled us to characterize perception for an ongoing neuroimaging study of the neural basis of size and distance perception (Maltseva, Quinlan, Stubbs, Konkle, & Culham, 2019).

Although pictorial cues and motion parallax are more informative for relative than absolute depth perception, vertical disparity can provide a cue to absolute distance (Brenner et al., 2001; Rogers & Bradshaw, 1993) for large surfaces (>20 degrees of visual angle; Bradshaw et al., 1996; Rogers & Bradshaw, 1995). Thus, it is possible that perception may have been closer to veridical in the binocular condition if the scene had included a large textured surface.

#### Why wasn't the FSE present in all conditions?

Some may be surprised that, although familiar size had strong effects in some conditions (e.g. up to a 1.5-cm difference in perceived size and a 15-cm difference in perceived distance between the Rubik's cube and die), it did not affect perception across all conditions equally. Specifically, FSEs on size perception were reduced for the conditions with extreme retinal angles (i.e. small far 1.3 degrees objects, which showed no significant FSE, and large near 15.9 degrees objects; see Figure 3d). This is consistent with evidence that participants take into account the relative range of stimuli presented, such that those with the largest relative retinal sizes are perceived as closer and those with the smallest—farther (Sousa et al., 2011; Sousa et al., 2012). Given that we used a within-subjects design, a complete set of trials exposed participants to our full range of retinal angles. However, past studies have found effects of retinal angles on perception even in between-subjects designs (Fitzpatrick et al., 1982; Gogel & Newton, 1969). Alternatively, our results may suggest that participants’ distance and size judgments are affected by absolute retinal angle, such that objects subtending large angles are perceived as nearer than those subtending small angles. In addition, the monocular pinhole condition had a potential confound: due to diffraction through monocular pinhole, the edges of the large near 15.9 degrees objects were not as sharp as those for other conditions. Although the contrast between the pinhole material and the background was low or negligible, if at all visible, it may have provided a relative size reference for all stimuli (Gogel & Newton, 1969).

#### Why was the FSE less than the physical size difference?

Even under the conditions in which we removed almost all visual cues to distance, and thus size, through monocular pinhole viewing and an intermediate (4.7 degrees) retinal angle, the largest FSEs we observed (1.5 cm) were less than the actual size difference between a Rubik's cube and a die (4.1 cm). Under these conditions, whereas the cues to the absolute distance of the objects are the same, there are multiple sources of relative depth information present within the objects, for instance linear perspective and texture foreshortening. Studies of size perception that use images often remove this cue by simply resizing images (see Mischenko; Figure 1); however, in our real objects, such information is available and may support estimates of object size and distance. Given that most studies of size constancy have used pictures or flat stimuli (like playing cards), the use of depth information internal to the object to support perceived size (and distance) is poorly understood.

#### Are FSEs specific to the perceptual measure used?

In contrast, the bulk of past studies on perceived size and distance, which typically had participants provide verbal reports about their percepts (Fillenbaum et al., 1965; Fitzpatrick et al., 1982; Gogel & Mertens, 1967; Gogel & Newton, 1969; Higashiyama, 1984; Schiffman, 1967), here, we used manual estimations of the percepts. This difference in reporting task is unlikely to account for the difference between our findings and earlier research. In fact, manual estimation not only has a reliability on par with other perceptual measures (Hartle & Wilcox, 2016), it may even be superior for estimations of familiar objects. Although verbal reports are largely biased by memory of responses on previous trials (Foley, 1980), manual estimation, being a kinematic task, is less likely to rely on explicit memory.

In the current study, manual estimation was performed without the hand or the object in view (i.e. in open loop); that is, vision was restricted as soon as the hand lifted off the table to perform the estimations (i.e. without a delay). Notably, the hand could not act as a relative size cue (Linkenauger, Leyrer, Bülthoff, & Mohler, 2013). Perceptual estimates based on an object that is no longer in view are thought to rely on a perceptual representation of the object held in memory, which may be less veridical than motor representations calculated on the fly (Goodale, Westwood, & Milner, 2004). Even though one may argue that observed FSEs could have been enhanced due to the visual system's reliance on memory of the object, FSEs have also been observed for reaching and grasping of concurrently viewed objects (i.e. action; visuo-motor procession is associated with the dorsal visual stream for action; McIntosh & Lashley, 2008).

Finally, one potential criticism of the experimental procedure is that short viewing durations may have inflated the FSE. Although viewing durations (*M* = 2.3 seconds, SD = 0.8 s) in the current study are, in fact, much shorter than unlimited viewing durations of most past studies (Fillenbaum et al., 1965; Franklin & Erickson, 1969; Gogel & Mertens, 1967; Ittelson, 1951; Ittelson & Ames, 1950), our participants self-determined the viewing time with a button press. The current viewing durations suggested participants did not scrutinize visual cues for an unlimited time (Epstein et al., 1961) but did take sufficient time to make relatively accurate perceptual judgments when possible, as indicated by high accuracy in the binocular viewing condition.

### Future directions

#### Behavioral studies

The current findings stimulate a number of possible questions to deepen understanding of the underlying nature of the potency of familiar size. While highly significant, the FSEs diminished under binocular viewing, when oculomotor cues were available. Although the contribution of pictorial cues and oculomotor cues are often discussed independently, pictorial cues may in fact influence the nature of the oculomotor cues. Pictorial cues may stimulate the visual system to verge and accommodate closer on objects that only appear closer due to the presence of pictorial cues on 2D displays (Batvinionak, Gracheva, Bolshakov, & Rozhkova, 2015). Due to the conflict between pictorial and oculomotor cues that 2D displays present, studying the interaction of these cues can be problematic. The current apparatus, in which real-world objects are presented with consistent binocular/oculomotor cues but in complete isolation from pictorial cues, offers a suitable paradigm to study interactions between pictorial and oculomotor cues. Going forward, the effect of other cues in an environment on the distance and size perception can be examined systematically. These include proprioceptive cues from the hand when positioned near the stimulus (Chen, Sperandio, & Goodale, 2018), vertical disparities, and relative size and distance compared with other objects in the scene. Another interesting topic for future research is how size and distance perception develop through infancy and childhood (DeLoache, Uttal, & Rosengren, 2004; Granrud et al., 1985; Sensoy, Culham, & Schwarzer, 2020). Recent evidence suggests that infants first come to apprehend familiar size for real objects with which they can interact (Sensoy, Culham, & Schwarzer, 2021).

#### Neural responses to familiar size

Our finding that familiar size has potent effects on perception, even when other disambiguating cues are present, resonates well with recent neuroimaging research. Growing evidence suggests that familiar size is a fundamental organizational principle within the ventral visual stream, which is specialized for visual perception and recognition. Some stimuli like faces, words, and small objects that typically subtend small retinal angles are processed largely within foveal vision; whereas other stimuli like scenes and large objects that typically subtend large retinal angles are processed over the full retina, including the periphery. Not only are these categories processed in different parts of the retina, they also appear to be preferentially processed in different parts of occipitotemporal cortex, with typically foveal stimuli processed in zones adjacent to the foveal representations of early visual areas and typically peripheral stimuli processed in zones adjacent to the peripheral representations of early visual areas (Hasson, Harel, Levy, & Malach, 2003; Konkle & Caramazza, 2013; Malach, Levy, & Hasson, 2002). Moreover, familiar-size selective responses can be evoked by relative size cues (Cate, Goodale, & Köhler, 2011) and can be learned (Coutanche & Thompson-Schill, 2019). Despite the evidence for cortical organization by familiar size, presentation distance can affect neural correlates of size perception in early visual processing (Chen, McManus, Valsecchi, Harris, & Gegenfurtner, 2019; Murray, Boyaci, & Kersten, 2006; Quinlan & Culham, 2007; Sperandio, Chouinard, & Goodale, 2012). Moreover, neuropsychological patients with visual agnosia recognize objects better when the physical size matches the familiar size (Holler et al., 2019). Ongoing work in our laboratory is examining the neural responses to the stimuli developed here in order to disentangle sensitivity to familiar size, physical size, physical distance, and retinal angle (Maltseva et al., 2019; Maltz, Stubbs, Quinlan, Rzepka, Martin, & Culham, 2020); the present behavioral results provide valuable data for modeling perceptual effects using representational similarity analysis (Kriegeskorte, Mur, & Bandettini, 2008).

## Conclusions

Our results show that familiar size is a potent visual cue that affects perceptual accuracy of size and distance of real objects, even when evident oculomotor cues to distance are available. Familiar size affects perception even for real objects when their physical dimensions can be computed on-the-fly from an immediate nonconflicting visual stimulation between oculomotor and pictorial cues (as opposed to images). Familiar size exerts a stronger effect on perceptual accuracy under conditions of greater uncertainty: when oculomotor cues are minimized (monocular pinhole viewing) and when retinal angle does not serve as a reliable cue to size and distance (when the retinal size is constant despite varying physical sizes and distances).
